# The President’s Address1Read at the Thirty-eighth Annual Meeting of the American Veterinary Medical Association, Hotel Rudolf, Atlantic City, N. J., September, 1901.

**Published:** 1901-10

**Authors:** Tait Butler

**Affiliations:** Raleigh, N. C.


### THE PRESIDENT'S ADDRESS.[1]

By Tait Butler,
RALEIGH, N. C.

You have just had striking examples of the tendency to comment on the marked progress made by the veterinary profession in recent years. In a general way we have all appreciated this progress, but it is doubtful if anyone here has fully grasped its scope and magnitude during even the last ten years. We have been so engrossed with the struggle to keep in touch with modern opinions and practice that we have had little time for retrospective contemplation.

We are also referred to as a young profession, which, when applied to the profession in America, is especially true. Even so young a man as your President has during his short life in the profession seen every existing veterinary college in the United States, with perhaps one exception, organized and developed to their present high state of efficiency. Our great national veterinary department, the Bureau of Animal Industry, which has solved more difficult problems and done more for the live-stock interests of this country than was ever done by any other similar institution in any country, came into existence about the time the speaker entered the profession. Whatever the veterinary profession in America is to-day has been the work of the last quarter of the nineteenth century. Not only is the profession, with its powerful and far-reaching influences for the good of the public health and wealth, a stalwart youth, but it is also largely the product of

[1] Read at the Thirty-eighth Annual Meeting of the American Veterinary Medical Association, Hotel Rudolf, Atlantic City, N. J., September, 1901.

the efforts of youthful men. I look before me in vain to find more than an occasional head sprinkled with gray, to denote long years of service in the cause. Perhaps no similar scientific association ever convened in such large numbers with so young and vigorous a personnel.

It is not my purpose to in any sense attempt a description of the matchless progress of the veterinary profession during the closing years of the last century. Such a task would be much more pleasant than the one I have essayed, but I see a duty in another direction. Permit me to say, however, that this progress has not always been direct and conservative, but sometimes, at least, rather meteoric and brilliantly erratic. Vigorous young manhood has its follies as well as its virility, and not, in all cases, has the progress of the profession been consistent and symmetrical. However, I am in no sense a pessimist. Indeed, in so far as the future of this Association and the veterinary profession is concerned, I am optimistic to a degree, but it seems proper to point out certain phases of work that have been neglected, and modestly criticise, where, in my judgment, faults exist that may be corrected.

Perhaps in no field of veterinary effort in America have better results been obtained than in veterinary college education. The three-year schools of to-day, with their longer periods for instruction and their laboratories and clinics, graduate men far superior to those of even fifteen or twenty years ago. Their graduates are better fitted to take proper rank in society, better equipped for serving the State as sanitarians, and better advisers on questions of animal hygiene and live-stock husbandry. They are better teachers of veterinary science as it is taught in the modern veterinary college; in fact, they are better qualified to perform very many of the duties required of them by existing conditions. They are doubtless better practitioners, judged from a purely scientific stand-point and from the stand-point of the college professor, who is accustomed to judge such matters by the artificial conditions of the college hospital and by the standards of city practice. But are they better qualified to earn their living as veterinary practitioners? I do not believe they are. I am not unaware that this statement is likely to meet with severe criticism, and before attempting to point out what seems to me the causes of this one-sided development of our veterinary education, it may be well to call your attention to two facts which should always be kept in mind when considering the nature and efficiency of veterinary college education. Regardless of the great furore which the colleges are creating over the opportunities for veterinarians as sanitarians, meat- and milk-inspectors, teachers of veterinary science in agricultural colleges, investigators for agricultural experiment stations, army veterinarians, and in all other similar capacities, it still remains a fact that nine out of every ten graduates must earn their living as general veterinary practitioners, and, therefore, fitness for general practice is the standard by which the efficiency of college education should be judged.

The other fact which may well be mentioned in this connection is that however important it may be that the veterinary practitioner should receive the highest possible scientific training, it nevertheless remains a fact that his success in practice depends more upon his ability to properly handle the owner of the patient than upon his ability to handle the patient itself. It, therefore, seems to me that the important question which the colleges should ask themselves is, Are we each year turning out men better able to earn their living and serve the country as general country practitioners? I am extremely doubtful if the graduate of to-day is able to give superior service under the conditions which, although undesirable, are nevertheless those which he must meet in ordinary country practice. The tendency of the colleges is to overlook the fact that the majority of veterinarians are not permitted to practice in the city or possess well-appointed hospitals. The result is that the conditions under which the undergraduate sees practice done at the colleges are very different from the conditions under which he must do practice if he is to succeed. In no line of business is there a greater need for developing a high type of that American characteristic of adaptability than in veterinary practice as it exists in this country at the present time. But as matriculate and graduate standards have been raised there has been a greater tendency for those better educated, but frequently less familiar with rural conditions, hence, less practical, to enter the profession. Therefore, the greater reason and necessity for more attention to the practical business side of veterinary education. There are too many teachers in our veterinary colleges who never had any experience in general, unaided, independent veterinary practice of any sort, and too many others whose experience has been one-sided, although, perhaps, extensive.

However, I think I see a more potent cause of the inability of the graduate of the present to adapt himself to the conditions of country practice than any of those yet mentioned. There has sprung up in recent years, especially among the more prominent members of the profession, a blind, unreasoning worship of all things European, especially German. American facts and methods and American investigators and literature fail to command the interest or respect of these great men, but German literature and investigators are followed and worshipped with a blind fatuity which would be ludicrous were it not fraught with serious consequences that imperil the future progress of the profession. "There are men who believe that there is culture in understanding the causes of the Trojan war, but none in learning the causes of the recent war in China." Just so with a large number of prominent American veterinarians. They appear to think a knowledge of German practice and literature the *sine qua non* of veterinary education, and exhibit the greatest contempt for American methods adapted to American conditions and needs. In all they say or do we are constantly reminded of the fact that the Germans do this or that, or that this or that particular practice or method is wrong because the Germans do not do it that way. Many of them do not even read American literature, or if they read it entirely discredit it, as evidenced by the fact that I read from the pen of one of these that anthrax is rare in this country but common in Europe. My friends, Drs. Dalrymple, of Louisiana, and Roberts, of Mississippi, and other Southern and Western veterinarians are probably convinced that anthrax is not particularly rare in this country. Again, an experiment station veterinarian informs his readers that the literature on a certain subject is "mostly foreign." Still, I find not less than a dozen bulletins treating the subject, besides numerous articles in veterinary journals and elsewhere. In all, probably double what he could find in the foreign literature of all languages. As a seeker after truth and a discoverer of abstract facts the German certainly has no superior, but he is in no sense the equal of the American in his application of facts to conditions, especially American conditions. The result is, we have numerous translations, but little strictly original literature from this class of men. Of these translations, which have been quite numerous in recent years, to which would you refer the young practitioner for aid in diagnosis or treatment? They are almost without exception absolutely worthless to the American country practitioner. They are written for other readers and apply to other conditions and business methods. When we have recovered from this mental dyscrasia and applied ourselves to the development of an American veterinary science suitable to the conditions existing in this country, we will see fewer failures in veterinary practice from what is now known as lack of practical "horse sense."

(To be continued.)

